# Kaposi’s sarcoma-associated herpesvirus infection promotes proliferation of SH-SY5Y cells by the Notch signaling pathway

**DOI:** 10.1186/s12935-021-02269-0

**Published:** 2021-10-30

**Authors:** Dongdong Cao, Shuyuan Wu, Xiaolu Wang, Ying Li, Huiling Xu, Zemin Pan, Zhaofu Wu, Lei Yang, Xiaohua Tan, Dongmei Li

**Affiliations:** 1 Key Laboratory of Xinjiang Endemic and Ethnic Diseases/NHC Key Laboratory of Prevention and Treatment of Central Asia High Incidence Diseases, School of Medicine, Shihezi University, Beier Road, Shihezi, Xinjiang China; 2 School of Medicine, Hangzhou Normal University, Hangzhou, Zhejiang China

**Keywords:** Kaposi's Sarcoma-associated herpesvirus, SH-SY5Y cell, Proliferation, Transcriptome sequencing, Notch signaling pathway

## Abstract

**Background:**

The cancer caused by Kaposi’s sarcoma-associated herpesvirus (KSHV) infection is one of the major causes of death in AIDS patients. Some patients have neurological symptoms, which appear to be associated with KSHV infection, based on the neurotropic tendency of this virus in recent years. The objectives of this study were to investigate the effects of KSHV infection on neuronal SH-SY5Y cells and to identify differentially expressed genes.

**Methods:**

KSHV was collected from islk.219 cells. Real-time PCR was used to quantify KSHV copy numbers. KSHV was used to infect SH-SY5Y cells. The KSHV copy number in the supernatants and mRNA levels of latency-associated nuclear antigen (LANA), ORF26, K8.1 A, and replication and transcriptional activator (RTA) were detected by real-time PCR. Proteins were detected by immunohistochemistry. The effect of KSHV infection on cell proliferation was detected by MTT and Ki-67 staining. Cell migration was evaluated by Transwell and wound healing assays. The cell cycle was analyzed by flow cytometry. The expression of CDK4, CDK5, CDK6, cyclin D1, and p27 were measured by western blotting. The levels of cell cycle proteins were re-examined in LANA-overexpressing SH-SY5Y cells. Transcriptome sequencing was used to identify differentially expressed genes in KSHV-infected cells. The levels of Notch signaling pathway proteins were measured by western blotting. RNA interference was used to silence Notch1 and proliferation were analyzed again.

**Results:**

SH-SY5Y cells were successfully infected with KSHV, and they maintained the ability to produce virions. KSHV-infected SH-SY5Y expressed LANA, ORF26, K8.1 A, and RTA. After KSHV infection, cell proliferation was enhanced, but cell migration was suppressed. KSHV infection accelerated the G0/G1 phase. CDK4, CDK5, CDK6, and cyclin D1 expression was increased, whereas p27 expression was decreased. After LANA overexpression, CDK4, CDK6 and cyclin D1 expression was increased. Transcriptome sequencing showed that 11,258 genes were upregulated and 1,967 genes were downregulated in KSHV-infected SH-SY5Y. The Notch signaling pathway played a role in KSHV infection in SH-SY5Y, and western blots confirmed that Notch1, NICD, RBP-Jĸ and Hes1 expression was increased. After silencing of Notch1, the related proteins and cell proliferation ability were decreased.

**Conclusions:**

KSHV infected SH-SY5Y cells and promoted the cell proliferation. KSHV infection increased the expression of Notch signaling pathway proteins, which may have been associated with the enhanced cell proliferation.

**Supplementary Information:**

The online version contains supplementary material available at 10.1186/s12935-021-02269-0.

## Background

Kaposi’s sarcoma-associated herpesvirus (KSHV), also known as human herpesvirus 8 (HHV-8), is the human oncogenic virus of Kaposi’s sarcoma (KS). KSHV infection is linked to other lymphoproliferative malignancies, including primary effusion lymphoma (PEL) and multicentric Castleman’s disease (MCD). Most reports on KSHV infection involve some aspect of endothelial cell and B lymphocyte function [1].

Acquired immunodeficiency syndrome (AIDS) patients are susceptible to KSHV infection [2], and some patients experience neurological symptoms such as memory loss, learning disabilities, and behavioral changes [3]. Little attention has been devoted to understanding how KSHV gains access to the central nervous system (CNS), thereby causing CNS symptoms. A few studies, which have suggested that KSHV has neuro-invasive potential, detected KSHV DNA in dorsal root ganglia of KS patients or in cerebrospinal fluid of HIV-positive patients by PCR [4, 5]. However, these reports could not unequivocally demonstrate the presence of KSHV in the brain parenchyma, becauseof extensive CNS vascularization. Furthermore, the known tropism of KSHV for immune lineages can yield in positive detection results.

KSHV is a double-stranded DNA virus with a genome length of about 170 kb [6]. The genome contains at least 90 open reading frames (ORFs), including 15 unique genes named K1 to K15; the others are represented by ORFs [7–9]. When host cells are infected with KSHV, the virus exhibits two phases, namely, persistent latent infection and transient lytic infection. Replication and transcription activator (RTA), which is encoded by ORF50, is the key switch and the regulatory protein controlling KSHV reactivation [6]. Gene expression during the latency phase mainly includes latency-associated nuclear antigen (LANA) encoded by ORF73, viral cyclin (v-cyclin) encoded by ORF72, viral FLIP (v-FLIP) encoded by ORF71, kaposin encoded by ORF-K12, and 25 mature viral miRNAs [10]. In the lytic state, the KSHV genome is changed to linear DNA, leading to virus production and virus spread. The virus expresses most genes in the lytic state, including ORF26, RTA, and K8.1 [11].

In a previous study, researchers combined immunohistochemical and immunofluorescent techniques with PCR to localize KSHV-infected cells in the brain parenchyma in asymptomatic HIV-positive individuals [12]. They showed that KSHV can infect human neurons, suggesting that the CNS could be a potential reservoir for KSHV. It remains to be determined whether KSHV infection can affect the function of cells in the CNS.

To understand the association between KSHV infection and CNS diseases, we infected SH-SY5Y cells with KSHV. Our results showed that KSHV could infect SH-SY5Y cells in vitro. We found that KSHV infection promoted the proliferation of SH-SY5Y cells and accelerated the G0/G1 phase by upregulating the expression of cyclins. We also identified differentially expressed genes by transcriptome sequencing and measured the expression levels of Notch1 signaling pathway proteins. In summary, this study reveals the effects of KSHV infection on neuronal cells and provides a theoretical basis for the therapeutic targeting of CNS diseases caused by KSHV infection.

## Results

### SH-SY5Y cells can be infected by KSHV

**Fig. 1:**
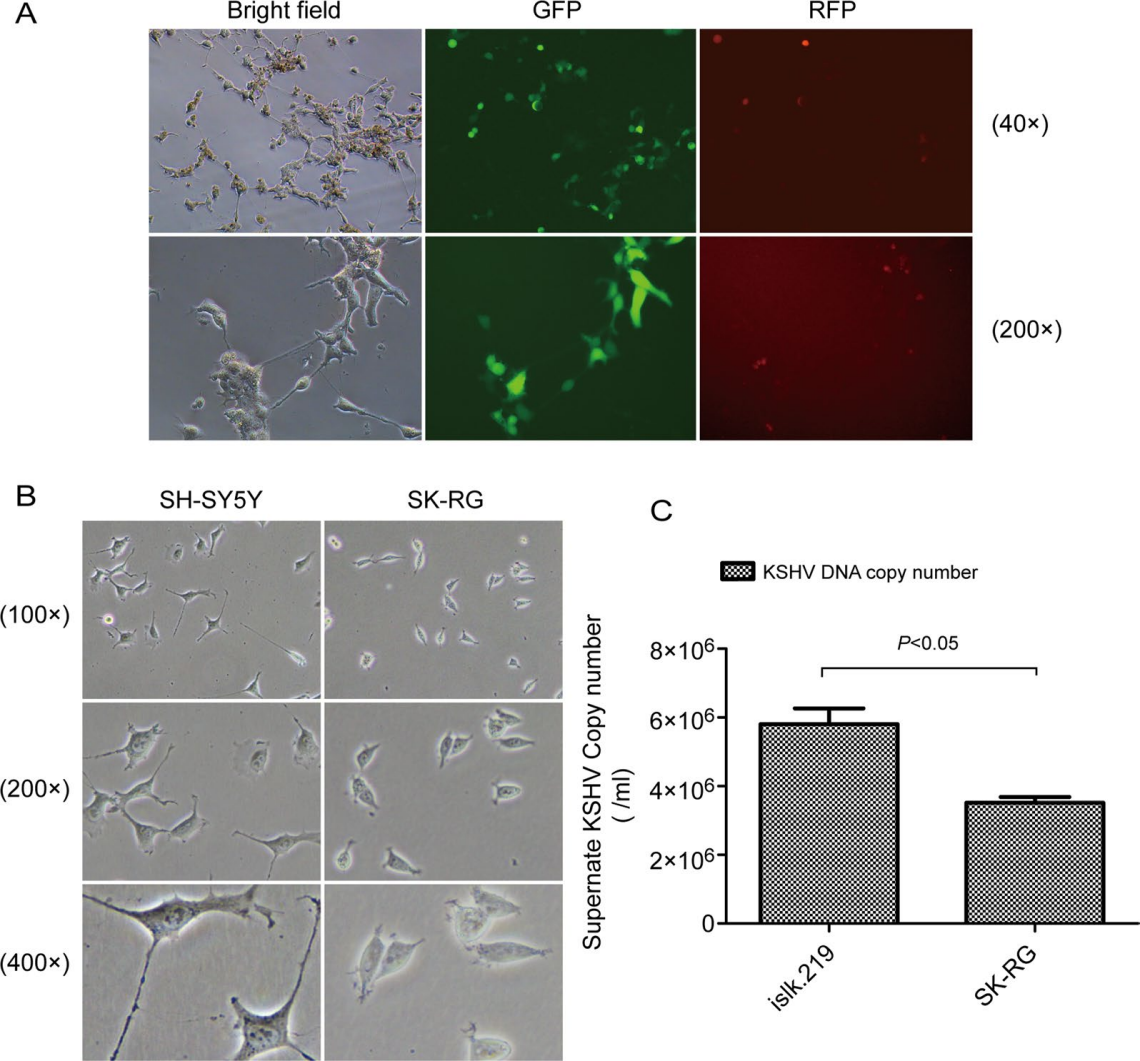
KSHV successfully infects SH-SY5Y cells. **A** Fluorescence microscope was used to confirm the successful infection. SH-SY5Y cells were infected with KSHV for 72 h and re-named as SK-RG cells. GFP fluorescence was used to confirm infection (magnification, ×40 and ×200). Some lytic state cells expressed RFP spontaneously. **B** The cell morphology was observed by microscope at ×100, ×200 and ×400. **C** The KSHV DNA copy number was detected by TaqMan real-time PCR. Supernatants were collected from lytic-induced islk.219 and SK-RG cells, after confirming infection, and cells were cultured for 7 days

To infect SH-SY5Y cells in vitro with KSHV, the virus was added to the supernatant of normal SH-SY5Y cells. Fluorescence detection was used to confirm infection. KSHV in islk.219 cells is a recombinant virus. It has an EF-1α promoter that expresses GFP to reflect the latent state and a PAN promoter that expresses RFP to reflect the lytic state. Infected cells expressed GFP. Some infected cells also expressed RFP at the same time (Fig. 1A). KSHV-infected SH-SY5Y cells were re-named as SK-RG cells. The morphology of SK-RG cells changed from aggregate-like to single-scatter growth. Cells became rounded and synapses were shorter (Fig. 1B). To determine if SK-RG cells could produce the virus, we collected the supernatants of lytic islk.219 and SK-RG cells, after confirming infection, and cultured these cells for 7 days. We extracted KSHV DNA. The KSHV copy number was (5.8 ± 0.6) × 10^6^ from the supernatant of induced islk.219 cells and (3.5161 ± 0.2) × 10^6^ from the supernatant of SK-RG cells (Fig. 1C). These results indicated that SH-SY5Y cells could be infected with KSHV and infected cells could release the virus into the supernatant.

### KSHV-infected SH-SY5Y cells express viral genes

**Fig. 2:**
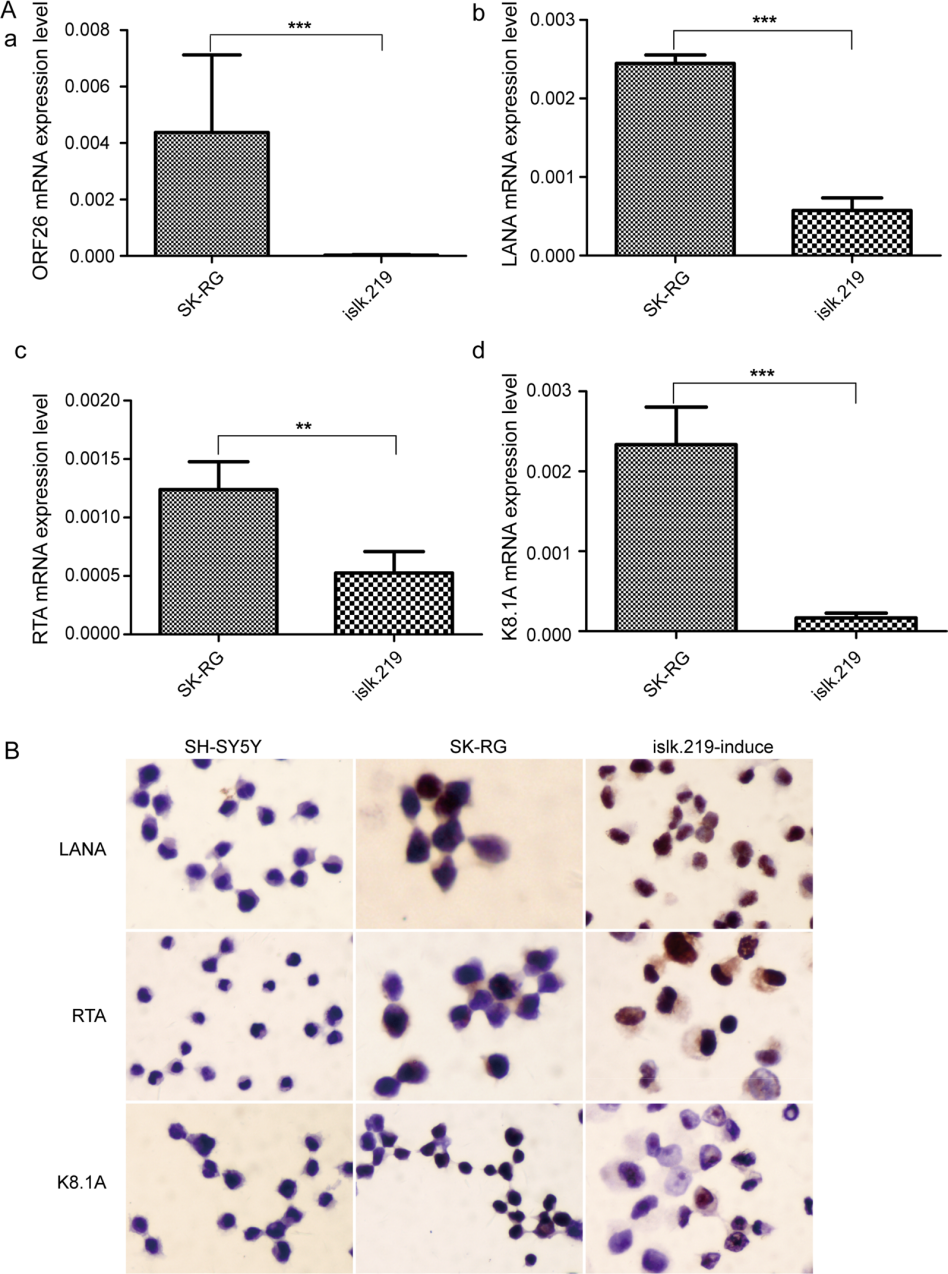
KSHV-infected SH-SY5Y cells express viral genes. **A** The mRNA expression level of KSHV genes in SK-RG cells were detect by real-time PCR, including the lytic genes, ORF26, RTA, K8.1 A, and the latent gene LANA. **B** The expression level of KSHV proteins in SK-RG cells were detected by IHC, including the lytic proteins, RTA, K8.1 A, and the latent protein LANA. Lytic-induced islk.219 cells served as the positive control, whereas SH-SY5Y cells served as the negative control. *P < 0.05; **P < 0.01; ***P < 0.001

We used real-time PCR to detect the mRNA levels of KSHV lytic genes, namely, ORF26, ORF50, K8.1 A, and latent gene LANA. SK-RG cells expressed all of these genes. We used islk.219 cells as the positive control (Fig. 2A). The protein levels of K8.1 A, RTA, and LANA were measured by IHC, with lytic islk.219 cells serving as the positive control and SH-SY5Y cells serving as the negative control. SK-RG cells and positive controls stained positively for K8.1 A, RTA, and LANA (Fig. 2B), indicating that KSHV-infected SH-SY5Y cells expressed viral genes.

### KSHV-infected cells have faster growth kinetics

**Fig. 3:**
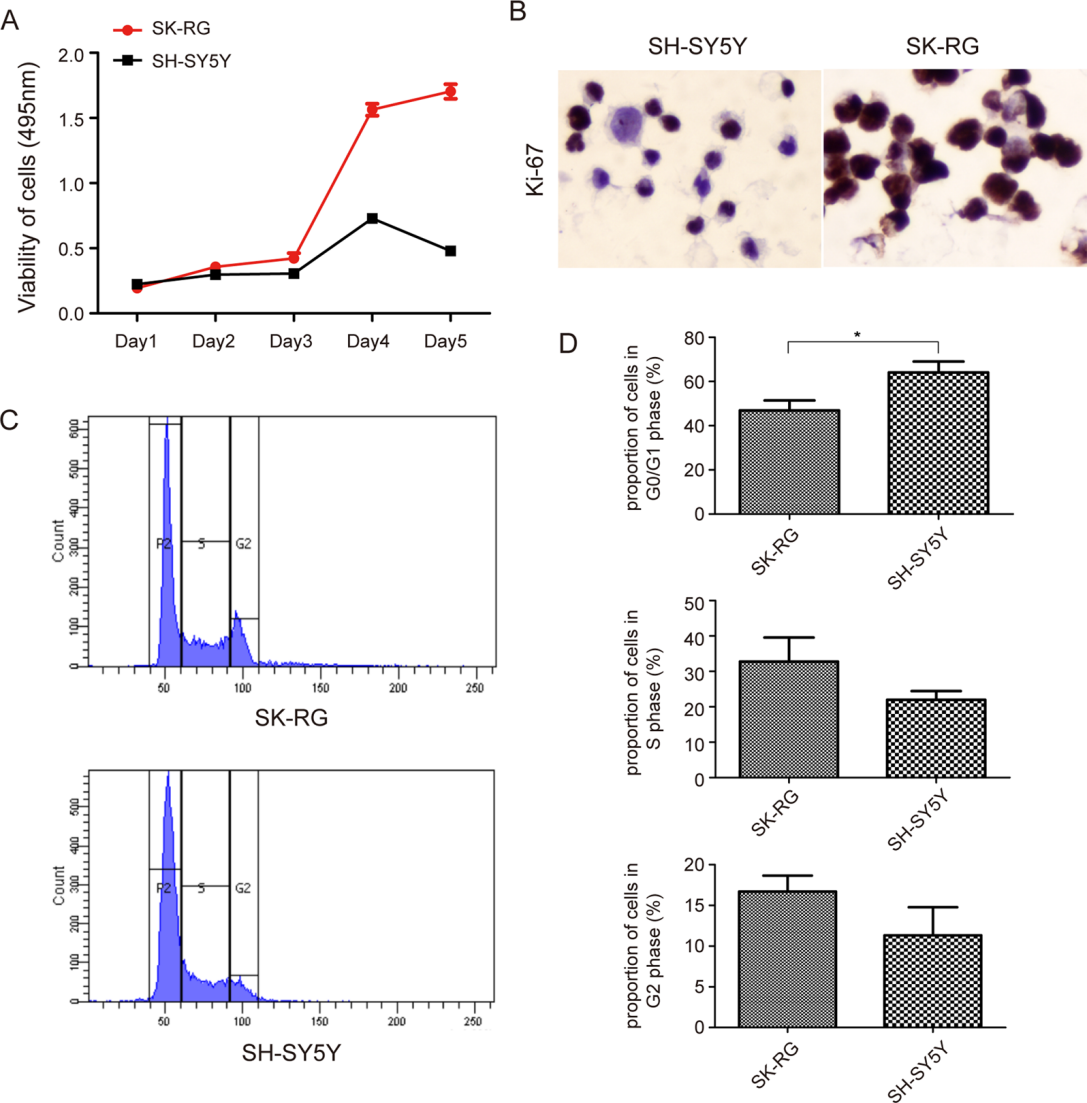
KSHV-infected SH-SY5Y cells have faster growth kinetics. **A** The effect of KSHV infection on cell viability was measured by MTT assay showing the growth of SK-RG cells to be faster than that of SH-SY5Y cells after 3 days (*P *< 0.05). **B** IHC was used to detect Ki-67 expression levels in SK-RG and SH-SY5Y cells. The results showing that the cells stained deeply in KSHV infected cells. **C**, **D** Using flow cytometry to detect SK-RG and SH-SY5Y cell cycles (1 × 10^6^), it was found that KSHV infection accelerated the G0/G1 phase (*P *< 0.05)

**Fig. 4:**
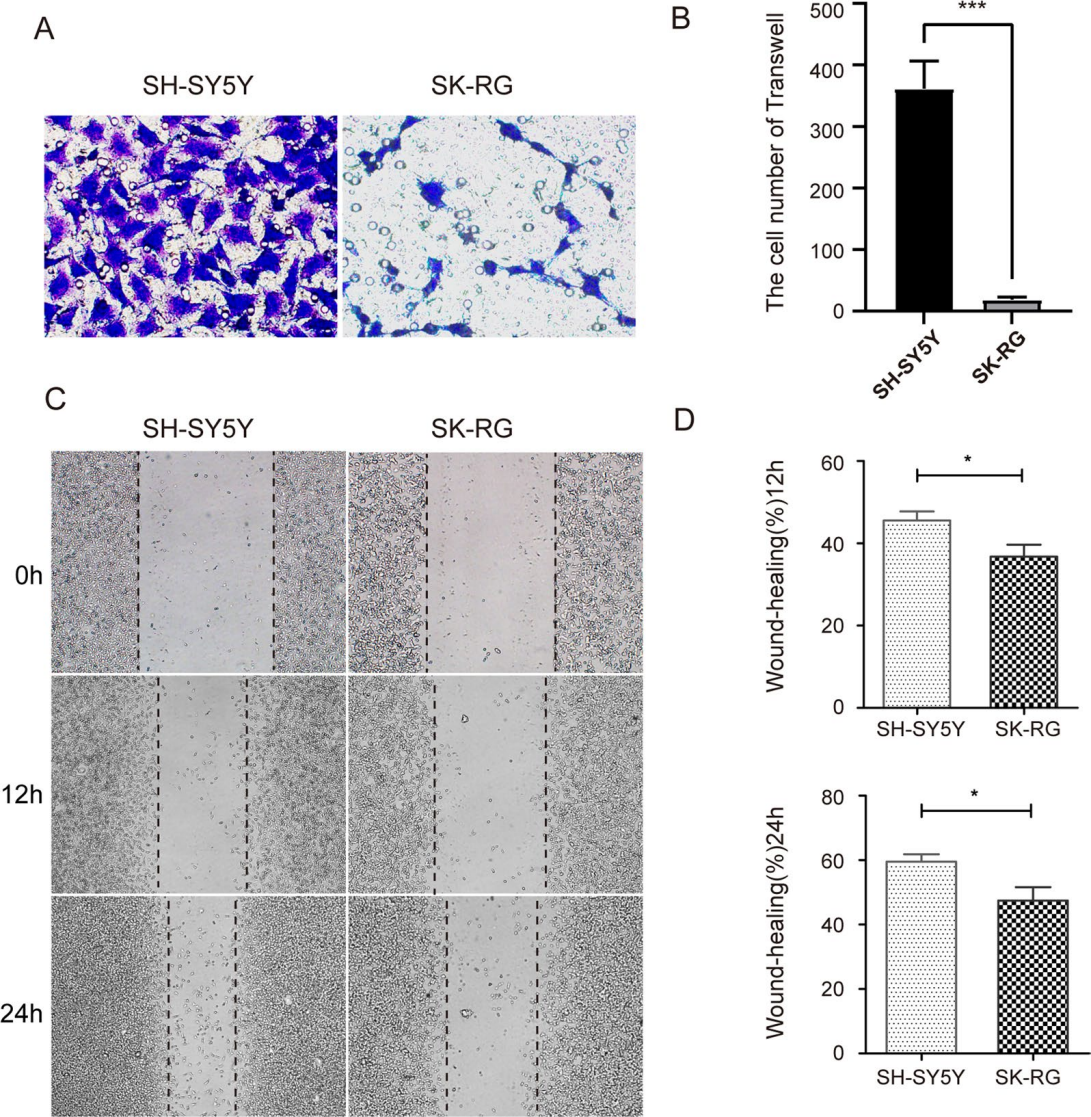
KSHV infection reduces the migratory ability. **A**, **B** Detection of the migratory ability of SK-RG and SH-SY5Y cells (2 × 10^4^) by transwell assay for 48 h. **C**, **D** Wound healing assay was used to detect the migratory ability of SK-RG and SH-SY5Y cells, and the results showing that the migratory ability of SK-RG cells was decreased compared with SH-SY5Y cells (*P *< 0.05)

The same number of SK-RG and SH-SY5Y cells were inoculated, and cell proliferation was assessed by the MTT assay. After 3 days, the absorbance was significantly higher for SK-RG cells than that for SH-SY5Y cells (*P *< 0.05) (Fig. 3A), revealing that KSHV infection promoted cell growth. We used IHC to detect the protein expression of Ki-67 in SK-RG and SH-SY5Y cells. Ki-67 stained strongly in SK-RG cells (Fig. 3B), indicating that KSHV infection promoted SH-SY5Y cell proliferation. The cell cycle plays a major role in cell proliferation. We examined the cell cycle in SK-RG and SH-SY5Y cells by flow cytometry. The proportion of G0/G1-phase SK-RG cells was lower than that of SH-SY5Y cells (*P *< 0.05) (Fig. 3C, D), demonstrating that KSHV-infected cells accelerated the G0/G1 phase. In the Transwell assay, the migratory ability of SK-RG cells to be lower than that of SH-SY5Y cells (Fig. 4A, B). In the wound-healing assay, the healing ability of SK-RG cells was lower than that of SH-SY5Y cells after 12 h of scratching (Fig. 4C, D). These results indicated that KSHV infection increased cell proliferation.

### KSHV infection increases expression of cyclins

**Fig. 5:**
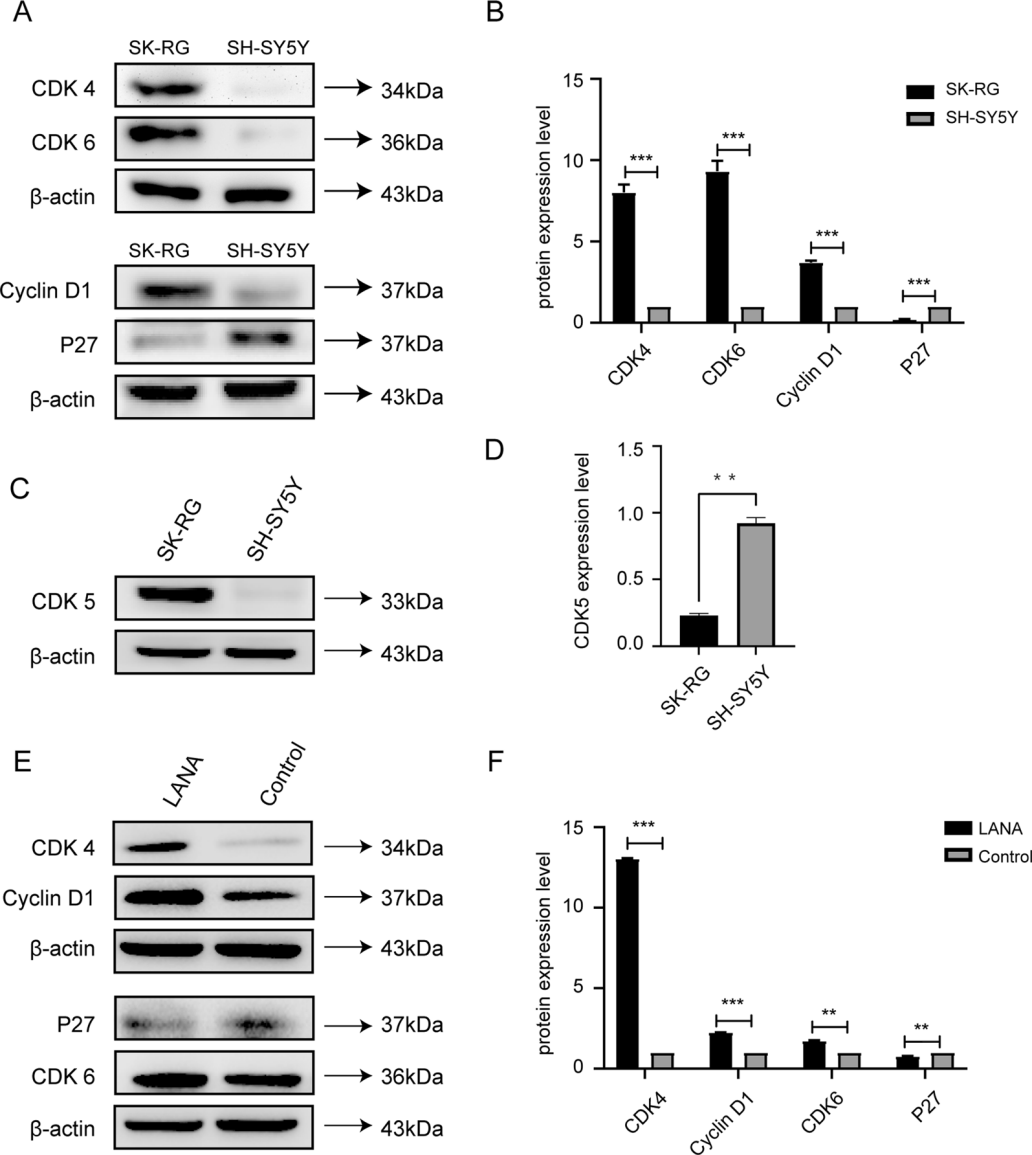
KSHV infection promotes the overexpression of cyclin proteins. **A**, **B** The expression level of CDK4, CDK6, cyclin D1 and p27 were detected by western blots in SK-RG and SH-SY5Y cells. **C**, **D** The expression level of CDK5 was detected by western blots in SK-RG and SH-SY5Y cells (*P *< 0.05). **E**, **F** Western blots was used to measure the expression level of CDK4, CDK6, cyclin D1 and p27 in SH-SY5Y cells before and after the LANA over expression (*P *< 0.001)

To further analyze the changes in cell cycle proteins, we used western blots to measure the expression levels of CDK4, CDK5, CDK6, cyclin D1, and p27. The expression of CDK4, CDK5, CDK6, and cyclin D1 was significantly increased in SK-RG cells compared with SH-SY5Y cells (Fig. 5A–D) (*P *< 0.05), whereas p27 expression was decreased compared with SH-SY5Y cells (*P *< 0.05).

The concept of virus pathogenicity indicates that the expression of host genes can be altered. LANA, an important KSHV gene, is involved in the replication of the cell cycle and the pathogenicity of the virus. We investigated the effect of LANA overexpression on cyclin expression. We overexpressed LANA in SH-SY5Y cells and measured the expression levels of CDK4, CDK6, cyclin D1, and p27. The expression levels of CDK4, CDK6 and cyclin D1 were significantly higher in the transfection group than those in the control group, the expression of p27 was significantly decreased in overexpressed cells compared with control cells (Fig. 5E, F).

### Differentially expressed genes were screened by transcriptome sequencing of SK-RG and SH-SY5Y cells

**Fig. 6:**
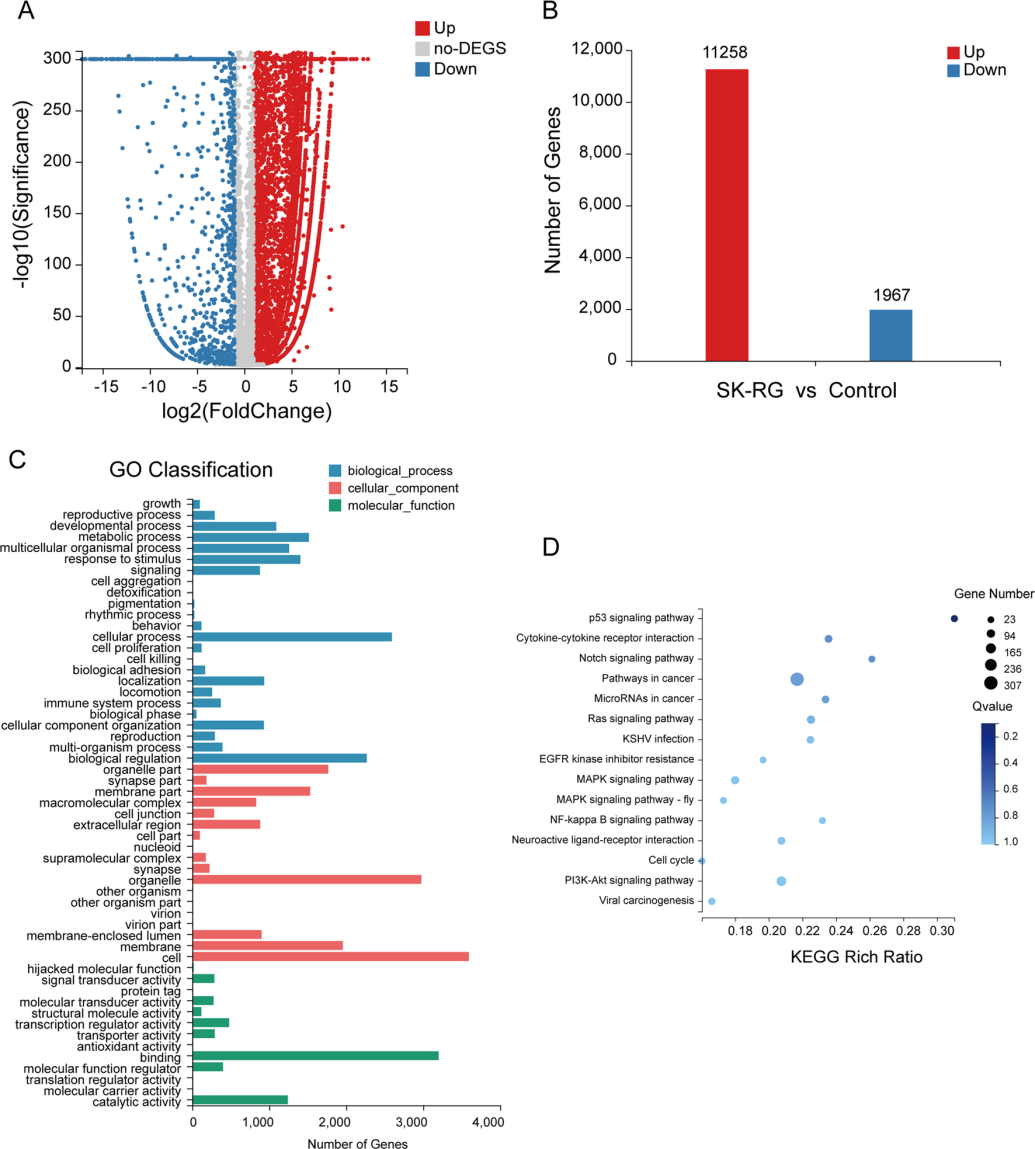
Transcriptome sequencing to identify differentially expressed genes in SK-RG and SH-SY5Y cells. **A** Based on the transcriptome sequencing, differentially expressed genes in SK-RG and SH-SY5Y cells, as shown by a volcano map. Red spots indicate up-regulated genes and blue spots indicate down-regulated genes in SK-RG cells. **B** RNA sequencing reveals differentially expressed genes. Compared with SH-SY5Y cells, 13,225 differentially expressed genes were detected in SK-RG cells, with 11,258 up-regulated genes and 1,967 down-regulated genes. **C** The results of GO analysis. GO analysis classified differentially expressed genes, as associating with molecular functions, cellular components, and biological processes. **D** KEGG pathway enrichment analysis results about the differentially expressed genes

For the analysis of differentially expressed genes in KSHV-infected SH-SY5Y cells, we used transcriptome sequencing. The datasets generated for this study can be found in the sequence read archive (SRA). The BioProject accession number is PRJNA627558. The SRA accession numbers for the three infected groups are SRR11596331, SRR11596330, and SRR11596329; those for the three uninfected groups are SRR11596328, SRR11596327, and SRR11596326. A total of 13,225 differentially expressed genes were detected in the SK-RG group (Fig. 6A). In the SK-RG group, 11,258 genes were upregulated and 1,967 genes were downregulated (Fig. 6B). GO analysis showed that based on molecular functions, the upregulated genes were primarily involved in cell binding, catalytic activity, and transcriptional regulation. Based on biological processes, the upregulated genes were primarily involved in cellular processes, biological regulation, and developmental processes (Fig. 6C). KEGG pathway analysis found that the Notch1 signaling pathway participated in the infection of SH-SY5Y cells by KSHV (Fig. 6D).

### KSHV infection increased the expression of Notch1 signaling pathway proteins

**Fig. 7:**
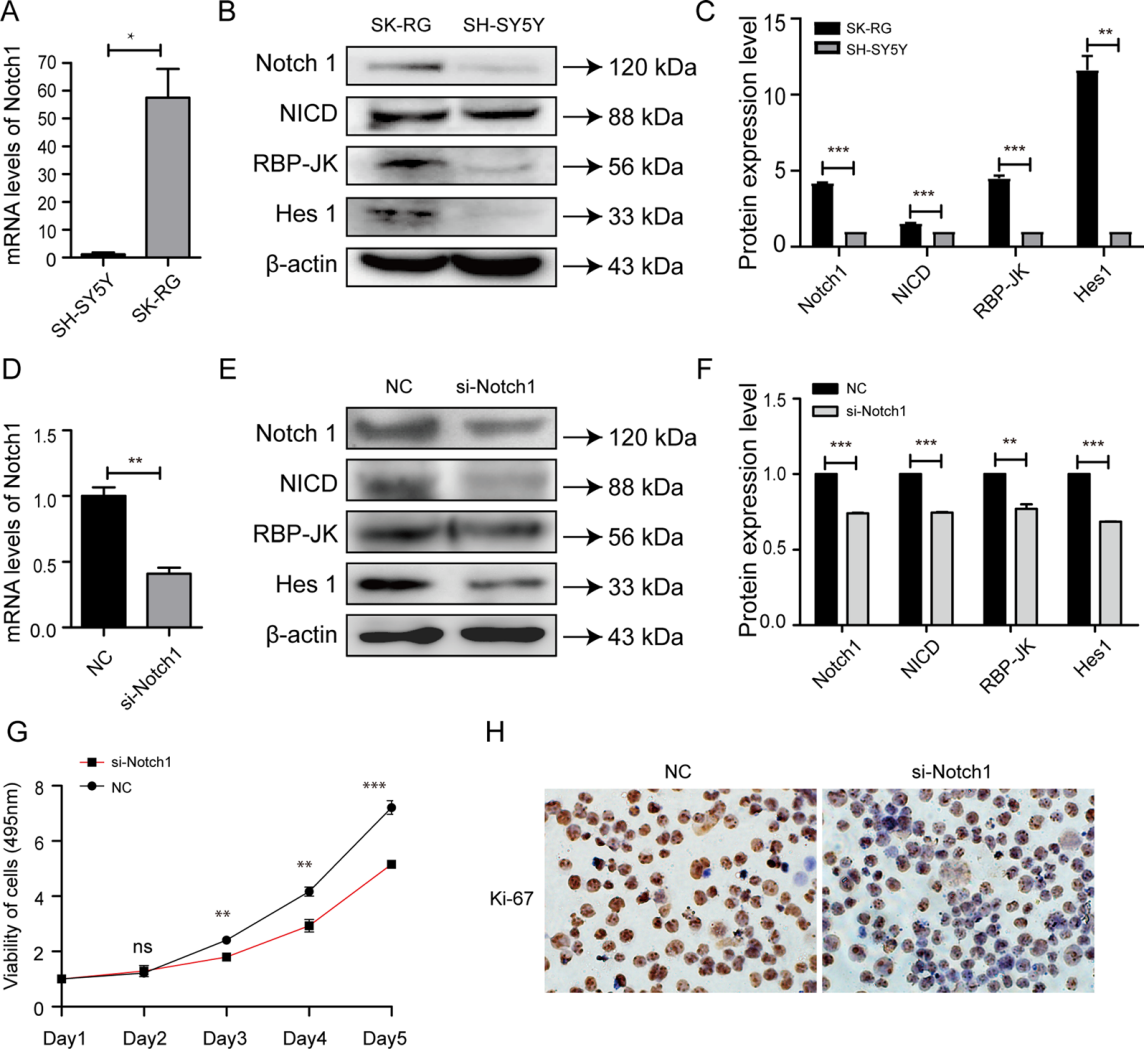
KSHV infection increased the expression of Notch 1 signaling pathway proteins and interfering Notch1 inhibits the proliferation of the cells. **A** Real-time PCR was used to analyze the mRNA expression level of Notch1 in SK-RG and SH-SY5Y cells. **B**, **C** The expression level of Notch1, NICD, RBP-Jĸ, and Hes1 were detected by western blots in SK-RG and SH-SY5Y cells. **D** RNA interference was used to interfere with the expression of Notch 1 in SK-RG cells, and then the efficiencies of Notch 1 interference was investigated using the real time-PCR. **E**, **F** Detection of Notch 1, NICD, RBP‐Jĸ, and Hes1 protein expression levels by western blot in si-Notch1 and NC group. **G** MTT assay was used to detect the proliferation ability in si-Notch1 and NC group. **H** The cell proliferation was detected by Ki-67 staining in si-Notch1 and NC group (*P* < 0.01)

According to KEGG pathway results, the Notch1 signaling pathway showed a strong correlation with infection. Therefore, we measured the expression level of Notch1 by real time-PCR and found that the mRNA expression level of Notch1 was higher in SK-RG cells than that in SH-SY5Y cells (Fig. 7A). We also measured the expression of Notch1 signaling pathway proteins, namely, Notch1, NICD, RBP-Jĸ, and Hes1 by western blots. The expression levels of Notch1, NICD, RBP‐Jĸ, and Hes1 were higher in SK-RG cells than those in SH-SY5Y cells (Fig. 7B, C), indicating that KSHV infection upregulated the expression of Notch1 signaling pathway proteins.Notch1 down-regulation inhibits the proliferation of SK-RG cells. In order to further explore the relationship between the Notch 1 signaling pathway and the proliferation of KSHV infected SH-SY5Y cells, we try to interfere with the expression of Notch1 and detect the relevant indicators of the SK-RG cell. We measured the expression level of Notch1 by real time-PCR to determine the interference result and found that the mRNA expression level of Notch1 was decreased in RNA interfering group than that in NC cells (Fig. 7D). We also measured the expression of Notch1 signaling pathway proteins, including Notch1, NICD, RBP‐Jĸ, and Hes1 by western blots. The expression levels of Notch1, NICD, RBP‐Jĸ, and Hes1 were higher in NC cells than those in RNA interfering group (Fig. 7E, F). The cell proliferation was assessed by the MTT assay. After 3 days, the absorbance was significantly higher for NC cells than that of RNA interfering group (*P *< 0.05) (Fig. 7F), revealing that interference with Notch1 inhibits cell proliferation. We further used IHC to detect the protein expression of Ki-67 in NC and RNA interfering group. Ki-67 stained strongly in NC cells (Fig. 7G), indicating that interference with Notch1 inhibits cell proliferation.

## Discussion

KSHV is an important member of the γ-herpesvirus family. The tropism of KSHV includes endothelial, epithelial, and some immune cell types such as B cells and macrophages [10].

Evidence indicates that human herpesvirus can enter the central nervous system through peripheral axons and the vascular system. Herpes simplex virus, human herpesvirus 4, and human herpesvirus 6 are associated with many neurological diseases, including primary central nervous system lymphoma, multiple sclerosis, Alzheimer’s disease, and cerebellar ataxia. A feature of this virus family is its ability to persist in human host cells indefinitely in a latent state [13–15].

HIV-positive patients are very susceptible to KSHV infection. In addition, 24% of HIV-positive patients have symptoms of aseptic meningitis [3]. As most herpesviruses exhibit some degree of neurotropism, studies have considered whether KSHV can infect the nervous system to cause dysfunction. Other studies have investigated whether KSHV can infect the nervous system. High levels of KSHV in paravertebral ganglia were reported in seven patients with AIDS who had Kaposi’s sarcoma [16]. Brink et al. [4] detected Epstein-Barr virus and KSHV DNA in the cerebrospinal fluid of patients infected with HIV using PCR and found that few patients were infected with KSHV. The neuroinvasive and neuropersistent potential of KSHV was implied from other studies. Said et al. [5] reported three cases with encephalitis, two of which were positive for HIV and harbored KSHV/HHV-8 sequences, as detected by PCR. Paul et al. [17] collected postmortem brain tissues from 30 patients for KSHV detection by PCR and found that 42 of 300 samples (14.0%) were KSHV positive. However, these studies did not directly prove that KSHV can infect neurons to cause CNS dysfunction.

To study the effects of KSHV in neuronal cells, we constructed a KSHV-infected cell model using SH-SY5Y cells, which were re-named as SK-RG cells. To infect SH-SY5Y cells, KSHV was collected from islk.219 cells. The KSHV in islk.219 cells is a recombinant virus. It has an EF-1α promoter that expresses GFP to reflect the latent state and a PAN promoter that expresses RFP to reflect the lytic state. Upon KSHV infection, the cells expressed GFP. If the virus is in the lytic state, the infected cells express RFP [18]. After KSHV infection, some infected SH-SY5Y cells steadily expressed both RFP and GFP without induction, indicating that KSHV can infect SH-SY5Y cells and spontaneously enter the lytic state.

To further expand these findings, we continuously detected the expression of KSHV genes. Latent KSHV expresses only a limited number of genes to evade the host cell’s immune response. The lytic virus expresses genes that are different from those of the latent virus [19]. We detected the mRNA of the latent gene LANA and those of lytic genes ORF26, RTA, and K8.1 A using real-time PCR. SK-RG cells expressed mRNA from both latent and lytic genes. We further detected the protein expression levels of LANA, RTA, ORF26, and K8.1 A using IHC, and the proteins were expressed in SK-RG cells. These results show that KSHV can infect SH-SY5Y cells, and some cells can express both latent and lytic genes.

In SK-RG cell cultures, we found that infected cells grew faster than non-infected cells. Therefore, we examined cell proliferation by MTT assay and Ki-67 staining. The results showed that proliferation was enhanced in KSHV-infected cells compared with uninfected cells. The manipulation of the cell cycle is a strategy commonly employed by viruses to achieve a favorable cellular environment during infection [20]. To examine cell proliferation, we used flow cytometry. The proportion of SK-RG cells in the G0/G1 phase was significantly lower than that of SH-SY5Y cells. The G0/G1 phase is the first phase of the cell cycle, in which cells organize prior to DNA replication. Any decisive event during the G0/G1 phase determines if the cell proceeds to division, pauses, or exits the cell cycle [21]. The cell cycle is also critical for tumorigenesis and tumor progression, and disruption of the cell cycle can reduce the stability of the genome and cause abnormal cell proliferation [22, 23].

Cyclins are involved in the cell cycle, and they activate cyclin dependent kinases (CDKs) [24]. Cyclin D1 is frequently deregulated in cancer, and it is a biomarker of tumor phenotype and disease progression. To investigate the effect of KSHV infection on the cell cycle, we measured the expression levels of cyclin D1, CDK4, CDK5, CDK6, and p27 by western blots. For instance, p27, an inhibitor of the cell cycle, is downregulated in the late G1 phase, allowing cells to complete the G1/S phase [25]. The expression of cyclin D1, CDK4, CDK5, and CDK6 in SK-RG cells was increased, whereas p27 expression was decreased in SK-RG cells, indicating that KSHV infection altered the cell cycle.

LANA is a major latent gene and transcription factor of KSHV [26]. Wei et al. [20] reported that LANA is an oncoprotein that steers KS and PEL cell cycle-related events, including cell proliferation and apoptosis by interacting with cellular and viral factors. LANA also promotes the proliferation of MCD [27] and endothelial cells [28]. We overexpressed the LANA plasmid in SH-SY5Y cells and examined the expression of CDK4, CDK6, cyclin D1, and p27. We found that LANA promoted the expression of cyclin D1, CDK4 and CDK6, which may explain why KHSV can promote the cell cycle and enhance cell proliferation.

The pathogenic mechanism of viruses involves an alteration of the expression of host genes. We screened for genes differentially expressed in SK-RG and SH-SY5Y cells using transcriptome sequencing. We found 11,258 upregulated and 1,967 downregulated genes in SK-RG cells compared with SH-SY5Y cells. According to KEGG pathway analysis, the Notch1 signaling pathway was activated in SK-RG cells and associated with KSHV infection. The Notch signaling pathway is composed of specific receptors (Notch1-4) and ligands (Delta-like 1, 3, 4, Jagged1 and Jagged2) in mammals [29]. The ligand-receptor interaction releasing the Notch intracellular domain (NICD) is carried out by the γ‐secretase complex. Subsequently, NICD translocates to the nucleus, where it binds to the DNA-binding factor recombinant binding protein suppressor of hairless (RBP‐Jĸ) and activates downstream factors such as Hes 1 and HEY [30–32].

The Notch signaling pathway is important in embryonic development, and in adult tissues, it regulates cell proliferation, differentiation, and apoptosis [33, 34]. Hou et al. [35] reported that overexpression of Notch1 promotes nasopharyngeal carcinoma cell growth. Du et al. [36] reported that Notch1 is overexpressed in gastric cancer and involved in cancer progression. The Notch signaling pathway is also critical for the development of KS. Curry et al. [37] reported that expression of Notch 1 is elevated in KS tumor cells. KSHV is reported to seize the Notch signaling pathway to inhibit other viruses in neighboring cells [38]. Zhuang et al. [39] demonstrated that Notch 1 acts as an activator of cell proliferation and a suppressor of cell apoptosis through the Akt/mTOR signaling pathway in renal cell carcinoma. Notch1 also mediates chemoresistance and strengthens the proliferative capacity of lung adenocarcinoma cells through the negative regulation of miR-451 by the transcription factor AP-1 [40]. Based on these reports, we examined the expression levels of Notch signaling pathway proteins, namely, NICD, RBP- Jĸ, and Hes1 in SK-RG and SH-SY5Y cells. We found that the expression levels of Notch1, NICD, Hes1, and RBP- Jĸ were significantly higher in SK-RG cells than those in SH-SY5Y cells.

In order to further clarify whether KSHV infection promotes the proliferation of SH-SY5Y cells by up-regulating the Notch1 signaling pathway, we used RNA interference to interfere with the expression of Notch1, and found that after interfering with Notch1, the expression level of Notch1 signaling pathway related proteins Notch1, NICD, Hes1, and RBP-Jĸ were reduced, and the proliferation ability of the cell is also suppressed, implying that the mechanism of KSHV pathogenicity involves the Notch1 signaling pathway.

We showed that KSHV can successfully infect SH-SY5Y cells in vitro. More importantly, we found that KSHV infection increases cell proliferation and accelerates the cell cycle of SH-SY5Y cells. The expression of CDK4, CDK5, CDK6, cyclin D1, and Notch signaling pathway proteins was upregulated, which can be helpful for studying the effects of KSHV on neuronal cell function.

In the future, we will investigate whether the increased growth of neuronal cells caused by KSHV infection can influence cell function such as their electrophysiological characteristics. Additionally, we will further examine the mechanism by which the Notch signaling pathway participates in KSHV infection in neuronal cells.

## Conclusion

KSHV can successfully infect SH-SY5Y cells in vitro and promote cell proliferation by upregulating the Notch signaling pathway.

## Methods

### Cell cultures

The SH-SY5Y cell line was cultured in Dulbecco’s modified Eagle medium (DMEM; Gibco, Grand Island, NY, USA) supplemented with 10% heat-inactivated fetal bovine serum (FBS; Hyclone, GE Healthcare, Little Chalfont, UK). The SH-SY5Y cell line was purchased from Shanghai Cell Bank, Chinese Academy of Sciences. KSHV-positive cells, namely, islk.219 cells, were a kind gift from Professor Ke Lan. In brief, islk.219 cells and KSHV-infected SH-SY5Y cells were cultured in the presence of 6 µg/ mL puromycin (Gibco), 100 µg/ mL G418 (Solarbio, Beijing, China), and 100 µg/ mL hygromycin (Invitrogen). All cells were incubated at 37 °C with 5% CO_2_.

### KSHV induction and virion collection

To induce KSHV lytic replication, 5 × 10^7^ islk.219 cells were treated with 1 µg/ mL doxycycline (Sigma-Aldrich, St. Louis, MO, USA) and 1.25 mM sodium butyrate (Sigma-Aldrich) for 72 h. The virus-containing supernatant was collected, and residual cells were removed by centrifugation at 3000 rpm for 15 min before filtering through a 0.45-µm filter. The same volume of prechilled PEG-it Virus Precipitation solution (System Biosciences, Shanghai, China) was added, and samples were stored at 4 °C overnight. The supernatant/PEG-it mixture was centrifuged at 1500×*g* for 30 min at 4 °C. The supernatant was aspirated, and the virus pellet was resuspended in sterile PBS. Virus stocks were stored at − 80 °C.

### Supernatant KSHV DNA extraction and counting

The supernatant was collected from KSHV-infected cells and treated with deoxyribonuclease I (DNase I) (ThermoFisher Scientific, Waltham, MA, USA) at 37 °C to remove residual genomic DNA. KSHV DNA was isolated using the phenol–chloroform extraction method. The same volume of phenol:chloroform:isoamyl alcohol (25:24:1) was added to the virus preparation, followed by centrifugation at room temperature for 5 min at 16,000×*g*. Thereafter, 1 µL of glycogen (20 µg/µL), 7.5 mol/L NH4OAc (0.5× volume of sample), and 100% ethanol (2.5× volume of sample + NH4OAc) were added, and samples were stored at − 20 °C overnight to precipitate the DNA. Samples were centrifuged at 4 °C for 30 min at 16,000×*g* to pellet the DNA, the supernatant was removed, and 150 µL of 70% ethanol was added. The sample was centrifuged at 4 °C for 2 min at 16,000×*g*, and the supernatant was collected. The DNA pellet was dried at room temperature for 10 min and resuspended in 100 µL of TEN buffer. The sample was centrifuged briefly to collect the KSHV DNA. The numble of KSHV DNA was counted using the TaqMan real-time PCR kits (Takara Biomedical Technology, Beijing, China). The amplification was carried out using primers listed in Table 1. The sensitivity was determined by testing decreasing DNA quantities of the ORF 26 plasmid (10-fold dilutions from 102 to 108 ng/µL). PCR was performed using cycling conditions as follows: 50 ℃ for 2 min and 95 ℃ for 10 min, followed by 40 cycles of 95 ℃ for 15 s and 60 ℃ for 1 min.

**Table 1:** List of oligonucleotide primers and probes

Target Gene	ID	Sequence (5′-3′)
Primer		
K8.1 A for real-time PCR	Forward	AAAGCGTCCAGGCCACCACAGA
	Reverse	GGCAGAAAATGGCACACGGTTAC
RTA for real-time PCR	Forward	GAGTCCGGCACACTGTACC
	Reverse	AAACTGCCTGGGAAGTTAACG
ORF26 for real-time PCR	Forward	CGAATCCAACGGATTTGACCTC
	Reverse	CCCATAAATGACACATTGGTGGTA
LANA for real-time PCR	Forward	AGCCACCGGTAAAGTAGGAC
	Reverse	AGCCACCGGTAAAGTAGGAC
Notch 1for real-time PCR	Forward	GAGGCGTGGCAGACTATGC
	Reverse	TCACGCTGACGGAGTACAAG
β-actin for real-time PCR	Forward	CGGAACCGCTCATTGCC
	Reverse	ACCCACATCGTGCCCATCTA
* ORF 26* for Taq-man real-time PCR	Forward	CGAATCCAACGGATTTGACCTC
	Reverse	CCCATAAATGACACATTGGTGGTA
Probe		
* ORF 26* for Taq-man real-time PCR		5′FAM/CCCATGGTCGTGCCGCAGCA/3′BHQ-1

### Infection of SH-SY5Y cells by KSHV

KSHV from islk.219 cells was used to infect SH-SY5Y cells at a density of 1 × 10^6^, with a KSHV virus copy number of 5 × 10^5^. Infected SH-SY5Y cells were cultured for 3 days. Green fluorescent protein (GFP) and red fluorescent protein (RFP) detection was performed using a fluorescence microscope (OLYMPUS, Tokyo, JPN), and infected SH-SY5Y cells were re-named as SK-RG cells.

### RNA extraction and real-time PCR

Total RNA from cells was extracted using TRIzol reagent (Invitrogen) according to the manufacturer’s instructions. RNA integrity was assessed by 1% agarose gel electrophoresis. 1 µg total RNA was reverse transcribed to cDNA using the Revert Aid First Strand cDNA Synthesis kit (ThermoFisher Scientific), following the manufacturer’s protocol, and each sample contained 1 µg of total RNA, which served as the template. The mRNA levels of K8.1 A, RTA, ORF26, LANA and Notch 1 were measured using the SYBR Green PCR kit (QIAGEN, Hilden, GER), following the manufacturer’s protocol and using primers listed in Table 1. The amplification protocol included an initial heat activation step at 95 °C for 5 min, followed by 35 cycles of 95 °C for 30 s and combined annealing/extension at 60 °C for 30 s. Results were calculated as 2^(−ΔCT)^ values, and the PCR reaction specificity was confirmed by melting curve analysis.

### Immunocytochemistry

Cells (2 × 10^6^) were centrifuged at 800 rpm for 5 min. The supernatant was aspirated, and cells were fixed with 4% paraformaldehyde for 20 min, centrifuged at 800 rpm for 5 min, washed with 1× PBS, and permeabilized with 0.1% Triton-X-100 for 15 min. The supernatant was removed, and cells were resuspended in 1× PBS. The cell solution was added drop-wise onto 12-well slides, with 10 µl per well. Slides were blocked overnight at 4 °C with antibodies that were diluted in Bond Primary Antibody diluent (Leica, Shanghai, China) as follows: anti-LANA (1:500, MBL, Japan), anti-RTA (1:400, ABBIOTEC, San Diego, CA, USA), and anti-K8.1 A (1:400, Santa Cruz Biotechnology, Santa Cruz, CA, USA). Slides were washed with 1× PBS and incubated with horseradish peroxidase-labeled polymeric secondary antibody. The Dako REALTM EnVision detection system (Dako, Glostrup, DK) was used for chromogen deposition, and hematoxylin was used for counterstaining.

### MTT assay

Cells (2 × 10^3^) were incubated in wells of a 96-well plate. After 1, 2, 3, 4, and 5 days of culturing, 20 µL of 3-(4,5-dimethylthiazol-2-yl)-2,5-diphenyltetrazolium bromide (MTT, Solarbio, Beijing, China) was added to each well. After 4 h at 37 °C in the dark, the MTT medium was removed and 100 µL of dimethyl sulfoxide (Solarbio) was added to each well. The absorbance was measured at an optical density of 490 nm (OD_490_)( BIO-RAD, California, CA, USA).

### Transwell and migration assays

A total of 2 × 10^4^ SK-RG and SH-SY5Y cells were seeded into 24-well plates housing 8-µm pore size chamber inserts. Cells were resuspended in 600 µL of serum-free DMEM and added into the upper chamber of each unit, whereas 20% FBS was added into the lower chamber before incubation at 37 °C in 5% CO_2_ for 48 h. After incubation, cells were fixed in methanol for 20 min and stained in 0.1% crystal violet for 30 min, followed by microscopic inspection. Six fields were randomly selected and cells were counted. This experiment was independently conducted three times.

Cell migration was assessed by the cell wound healing assay. SH-SY5Y and SK-RG cells (1 × 10^6^) were cultured in 6-well plates for 24 h. When the cells reached a confluency of about 80%, we used a sterile 1-mL pipette tip to scratch the monolayer, washed the cells twice with PBS, and incubated the cells in DMEM supplemented with 2% serum for further culture. Cells were photographed using a microscope at 0, 12, and 24 h after scratching at an identical location.

### Flow cytometry

SK-RG and SH-SY5Y cells (1 × 10^6^) were digested with trypsin without EDTA and centrifuged at 800 rpm for 5 min. Cells were fixed in ice-cold methanol and stored at − 20 °C overnight. Cells were centrifuged at 800 rpm for 5 min and resuspended in 500 µL of 1× PBS. Thereafter, 5 µL of PI and 5 µL of RNAse A were added, cells were incubated at 37 °C in the dark for 30 min, and the cell cycle was examined by flow cytometry (BD Biosciences, San Jose, CA, USA).

### Transcriptome sequencing

Transcriptome sequencing was performed by BGI Shenzhen Company using three independent batches of SK-RG cells and three independent batches of SH-SY5Y cells. The samples were analyzed using the BGISEQ-500 platform and filtered using SOAP nuke software (BGI Shenzhen Company). After obtaining clean reads, hierarchical indexing was used for spliced alignment of transcripts to compare the clean reads to the reference genome sequence, and the integrative genomics viewer was used to browse the alignment of reads with the genome.

BGI confirmed the differentially expressed genes as previously described [41]. The DEGseq method, which is based on the Poisson distribution, was used for data normalization and screening of differentially expressed genes. To remove low-repeat differentially expressed genes, the *P*-value was required to be less than 0.05. Fold-changes were defined as >0 for up-regulated genes and <0 for down-regulated genes.Horizontal clustering and gene ontology (GO) were used to calculate the number of genes in different nodes. Pathway enrichment analysis of differentially expressed genes used the Kyoto Encyclopedia of Genes and Genomes (KEGG) database.

### Western blots

Proteins were fractionated by sodium dodecyl sulfate polyacrylamide gel electrophoresis, transferred to polyvinylidene difuoride (PVDF) membranes (Millipore,Shanghai, China),. PVDF membranes were blocked with 5% nonfat milk at room temperature for 2 h, and incubated with antibodies at 4 °C overnight,as described previously [42]. Primary antibodies were used as follows: anti-CDK4 (1:1000, Bioward Technology, Nanjing, China), anti-CDK5 (1:1000, Boster, Wuhan, China), anti-CDK6 (1:500, Bioward Technology), anti-cyclin D1 (1:500, Bioward Technology), anti-p27 (1:1000, Bioward Technology), anti-Notch1 (1:1000, Cell Signaling Technology, Boston, MA, USA), anti-NICD (1:1000, Abcam, Cambridge, MA, USA), anti- RBP-Jĸ (1:1000, Abcam), anti-Hes1 (1:1000, Cell Signaling Technology), and anti-β-actin (1:1000, ZSGB-BIO, Beijing, China). The secondary antibody was goat anti-mouse IgG (1:10,000, ZSGB-BIO) or goat anti-rabbit IgG (1:10,000, ZSGB-BIO), and enhanced chemiluminescence (ECL) detection (Millipore, Temecula, CA, USA). The exposure was performed using the Flour Chem HD2 imaging analyzer from ProteinSimple company.

### Transfection and RNA interference assays

The LANA plasmid was obtained from Hangzhou Normal University. 1 × 10^6^ SH-SY5Y cells were transfected with 2 µg of the LANA plasmid or the empty vector using Lipofectamine 2000 transfection reagent (Invitrogen) according to the manufacturer’s instructions. After 24, 48, and 72 h, cells were lysed, and RNA was extracted for real-time PCR to determine the transfection efficiency (Additional file 1: Fig. S1). The interference sequence of Notch1 (Gene ID: 4851) was synthesized by Shanghai Jima Pharmaceutical Technology Co., Ltd., and the sequence is: 5′-AAGGUGUCUUCCAGAUCCUGA-3′. The negative control (NC) sequence is: 5′UUC UCC GAA CGU GUCACG UTT-3′. SK-RG cells were transfected with 2 µg si-Notch 1 or NC using Lipofectamine 2000 transfection reagent (Life Technologies) according to the manufacturer’s instructions.

#### Statistical analysis

Statistical analysis was performed using SPSS version 20.0 software (SPSS Inc., Chicago, IL, USA). Independent *t*-tests were used for data measurement. Error bars represented standard deviations. *P *< 0.05 was considered statistically significant.

## Supplementary Information

**Additional file 1: Fig. S1.** Efficiency of LANA plasmid transfection. After 1, 2, and 3 days, the transfection efficiency of LANA was examined by real-time PCR.

## Data Availability

The datasets used and/or analyzed in the current study are available from the corresponding author upon reasonable request.

## Abbreviations

KSHV: Kaposi's sarcoma-associated herpesvirus
GFP: Green fluorescent protein
LANA: Latency-associated nuclear antigen
ORF: Open reading frame
RTA: Replication and transcription activator
MTT: 3-(4,5-dimethylthiazol-2-yl)-2,5-diphenyltetrazolium bromide
CDKs: Cyclin-dependent kinase
NICD: Notch intracellular domain
RBP-Jκ: Recombination signal sequence-binding protein J kappa
HHV-8: Human herpesvirus 8
KS: Kaposi's sarcoma
PEL: Primary effusion lymphoma
MCD: Multicentric Castleman’s disease
AIDS: Acquired immunodeficiency syndrome
CNS: Central nervous system
v-cyclin: Viral cyclin
v-FLIP: Viral FLIP
DMEM: Dulbecco’s modified Eagle medium
FBS: Fetal bovine serum
DNase I: Deoxyribonuclease I
RFP: Red fluorescent protein
GO: Gene ontology
KEGG: Kyoto Encyclopedia of Genes and Genomes
