# Supplementary figures and images for: Kaposi’s sarcoma-associated herpesvirus infection promotes proliferation of SH-SY5Y cells by the Notch signaling pathway
Source: Cancer Cell Int. 2021 Oct 30;21:577. doi: 10.1186/s12935-021-02269-0 (PMC8557577; doi:10.1186/s12935-021-02269-0)

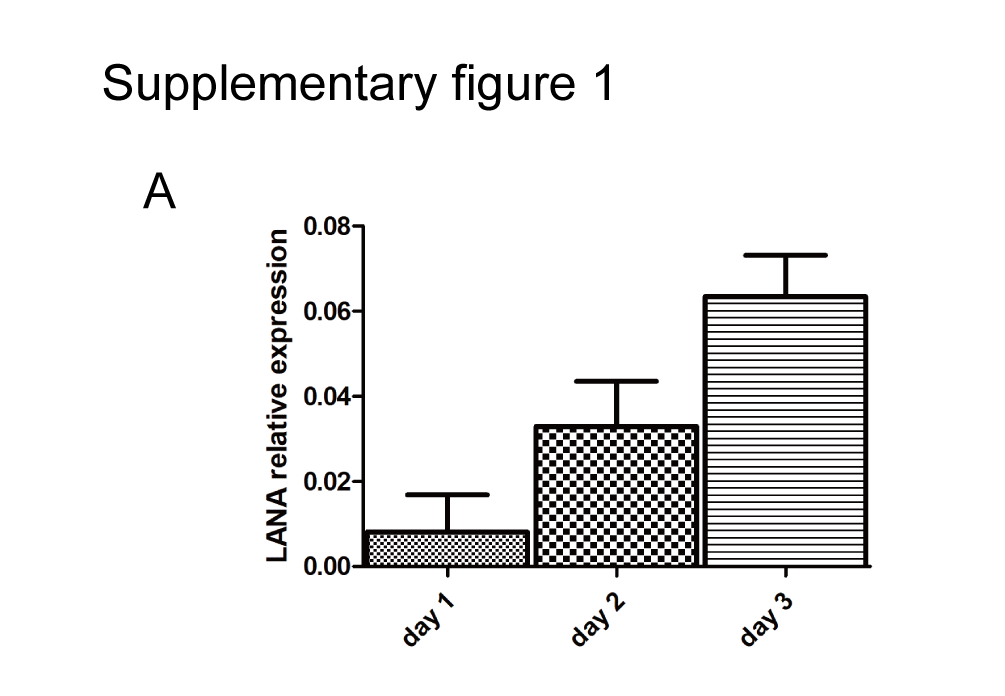

Supplement: Supplementary file 1 — Additional file 1: Fig. S1. Efficiency of LANA plasmid transfection. After 1, 2, and 3 days, the transfection efficiency of LANA was examined by real-time PCR. [file 12935_2021_2269_MOESM1_ESM.tif]
